# Bacteria Getting into Shape: Genetic Determinants of *E. coli* Morphology

**DOI:** 10.1128/mBio.01977-16

**Published:** 2017-03-07

**Authors:** Shawn French, Jean-Philippe Côté, Jonathan M. Stokes, Ray Truant, Eric D. Brown

**Affiliations:** a Department of Biochemistry and Biomedical Sciences, McMaster University, Hamilton, Ontario, Canada; b Michael G. DeGroote Institute for Infectious Disease Research, McMaster University, Hamilton, Ontario, Canada; University of Rochester

## Abstract

Perturbation of cellular processes is a prevailing approach to understanding biology. To better understand the complicated biology that defines bacterial shape, a sensitive, high-content platform was developed to detect multiple morphological defect phenotypes using microscopy. We examined morphological phenotypes across the *Escherichia coli* K-12 deletion (Keio) collection at the mid-exponential growth phase, revealing 111 deletions perturbing shape. Interestingly, 64% of these were uncharacterized mutants, illustrating the complex nature of shape maintenance and regulation in bacteria. To understand the roles these genes play in defining morphology, 53 mutants with knockouts resulting in abnormal cell shape were crossed with the Keio collection in high throughput, generating 1,373 synthetic lethal interactions across 1.7 million double deletion mutants. This analysis yielded a highly populated interaction network spanning and linking multiple phenotypes, with a preponderance of interactions involved in transport, oxidation-reduction, and metabolic processes.

## IMPORTANCE

Genetic perturbations of cellular functions are a prevailing approach to understanding cell systems, which are increasingly being practiced in very high throughput. Here, we report a high-content microscopy platform tailored to bacteria, which probes the impact of genetic mutation on cell morphology. This has particular utility in revealing elusive and subtle morphological phenotypes associated with blocks in nonessential cellular functions. We report 111 nonessential mutations impacting *E. coli* morphology, with nearly half of those genes being poorly annotated or uncharacterized. Further, these genes appear to be tightly linked to transport or redox processes within the cell. The screening platform is simple and low cost and is broadly applicable to any bacterial genomic library or chemical collection. Indeed, this is a powerful tool in understanding the biology behind bacterial shape.

## OBSERVATION

Bacterial cell shapes represent billions of years of evolutionary refinement. Their small size results in high surface area-to-volume ratios, which are ideal for nutrient transport, efficient respiration, and maintenance of structural integrity. Overall cell shape is known to be governed by a wealth of cellular processes and environmental cues (1–3). It quickly becomes challenging, however, to pinpoint how variations in shape impact cell function. Genome-scale data describing genetic contributions to bacterial morphology are a step toward addressing this question. Further, the resulting interaction networks represent multiple linked biological networks arising from different shape phenotypes. Herein, we describe a genomic snapshot of *Escherichia coli* K-12 morphology at the mid-exponential phase using the Keio deletion collection (4). We further characterize genetic interactions between deletions impacting shape and the *E. coli* genome using high-throughput conjugation (5, 6).

**FIG 1:**
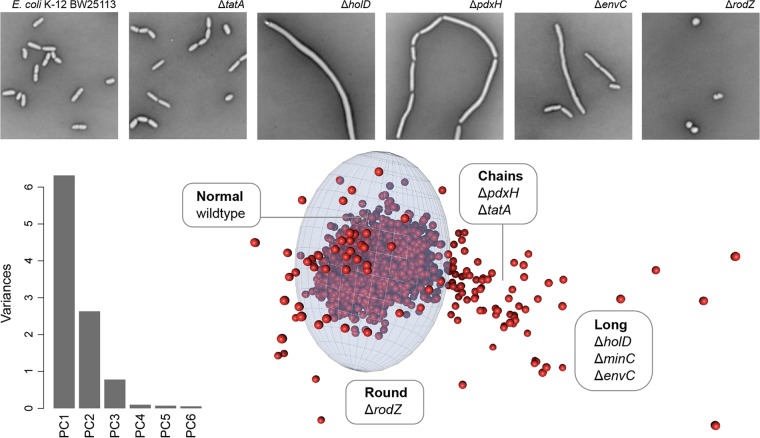
High-throughput cell shape screen of the *E. coli* nonessential gene deletion (Keio) collection. The micrographs shown were captured in a high-throughput modification to the microscopic methods described by Czarny et al. (25). The top panels represent example morphological phenotypes observed in genes from the Keio collection: wild-type *E. coli* K-12 BW25113 cells, Δ*tatA* long and chained cells, Δ*holD* very long cells, Δ*pdxH* long and occasionally chained cells, Δ*rodZ* round cells, and Δ*envC* long cells. An annotated data set is provided in Table S1, where individual gene hits can be better visualized. The bar graph shows principal-component analysis of features from the image analysis and reveals that most of the variance is explained by three principal components. When plotted in three dimensions, these principal components can be fit with an ellipsoid surrounding an origin that describes the variation of cell morphologies. The ellipsoid is bounded by distances from the origin that are 3 standard deviations in each principal-component dimension, respectively, from the origin describing wild-type cells. Shape-perturbing mutations are defined here as having principal-component coordinates outside the ellipsoid.

The high-content imaging platform that we developed is highly tailored for the complicated task of bacterial imaging. Imaging times for high-density (384- or 1,536-well) microwell plates can far exceed the approximately 20-min doubling time for *E. coli*, presenting temporal challenges to imaging genomic libraries within a common phase of growth. Further, the motility of live bacterial cells presents additional difficulties in limiting the mobility of cells during microscopic imaging. We countered these challenges by utilizing a glutaraldehyde fixative; a common first step in electron microscopy preparations and known to preserve cell shape with negligible impacts at the resolution of light microscopy (7). Cells were then negatively stained in glass bottom imaging plates (0.17-mm thickness) using nigrosin and gently heat fixed with humidity control, bringing all cells to a common focal plane. This obviated the need for phase-contrast, which was unworkable due to the light cone from the phase annulus being blocked by well edges in 384-well and higher-density microplates. Using a bright field allowed for more light to pass through the sample, resulting in faster exposure and better focal accuracy. Plates prepared in this manner can be reimaged indefinitely, and image quality does not degrade with time, facilitating high-content imaging of samples that have been fixed in a specific growth phase. The resulting images display intense contrast between cells and background, which is highly desirable for quantitative imaging (Fig. 1; fully detailed in Text S1 and Fig. S1 in the supplemental material). Ten morphological features were quantified per knockout: area, perimeter, minimum ellipse axis, minimum Feret length, major ellipse axis, major Feret length, circularity, roundness, aspect ratio, and solidity. We then made use of principal-component analysis (PCA) as a multiparametric analysis and means of dimensionality reduction (Fig. 1; Fig. S1). This gave us a completely unbiased means of phenotypic sorting after acquisition. Abnormally shaped treatments were revealed by fitting an ellipsoid around the cluster of data representing wild-type morphology in principal-component space and selecting for those falling outside this cutoff zone. The *x*, *y*, and *z* boundaries of the ellipsoid in each dimension correspond to a 3-standard-deviation cutoff in the first three principal components.

10.1128/mBio.01977-16.1 TEXT S1  In-depth methods for the microscopy pipeline and the downstream genetic array creation. Download TEXT S1, DOCX file, 0.1 MB. Copyright © 2017 French et al. 2017 French et al. This content is distributed under the terms of the Creative Commons Attribution 4.0 International license.

10.1128/mBio.01977-16.4 FIG S1  Overall pipeline for micrograph feature extraction. Shown here as an example are wild-type *E. coli* K-12 BW25113 cells after our fixation and staining method has been applied (a). Alongside the wild-type raw image is a cross section of a single cell, highlighting the intense pixel contrast between the cell and the background. Thresholding of the image using the Otsu algorithm results in a Boolean image (b), to which a watershed algorithm is applied to produce single cells (c), even within large adjacent clusters. Outlined in panels d to g are the density distributions of various extracted features within several strains. In black is a wild-type culture, in blue is a Δ*rodZ* culture, and in red is a Δ*pdxH* culture. The distributions are broad, even within individual cultures: this is expected given the heterogeneous sizes, especially in filamentous strains such as the Δ*pdxH* mutant, evident in panel e. There is clear distinction between the treatments, however, especially when a multivariate approach is used, such as in Fig. 1. Download FIG S1, PDF file, 0.7 MB. Copyright © 2017 French et al. 2017 French et al. This content is distributed under the terms of the Creative Commons Attribution 4.0 International license.

In this manner, we identified some 111 deletion mutants with shape defects (see Table S1 and Data Set S1 in the supplemental material). Our approach was validated with the confirmation of several known shape mutants, including the *tatA* deletion mutant, previously shown to be long and chained due to improper localization of amidases in the periplasm (8). Mutations in *holD*, encoding the DNA polymerase III ψ subunit, are known to cause filamentous morphologies in *E. coli* (9). Indeed, DNA replication has a synchronous relationship with cell division processes (10, 11), with disruptions in DNA gyrase resulting in elongated cells with single chromosomes and lacking septa (12). Interestingly, pyridoxine 5′-phosphate oxidase, encoded by *pdxH*, generates pyridoxal 5′-phosphate (PLP) and caused a shift toward long, occasionally chained cells. PLP is a key cofactor in the provision of d-alanine and d-glutamate for cell wall peptidoglycan synthesis (13), and *pdxH* mutants are known to have a filamentous phenotype (14). Similarly, EnvC is an activator of cell wall remodeling enzymes and is known to cause filamentous growth (15), and RodZ is involved in sidewall formation and is known to cause spherical morphology (16). These phenotypes were all confirmed in our screen. Surprisingly, 52 genes in our list encode uncharacterized proteins in *E. coli*, with many being putative outer or cytoplasmic membrane proteins (Table S1). Thus, the high-content screening platform described identified nonessential genes involved in maintaining shape. To further characterize and probe biological processes interacting with these mutations, we opted to prepare a genetic interaction array with a focus on these shape genes.

10.1128/mBio.01977-16.7 TABLE S1  Gene knockouts in *E. coli* that confer an abnormal shape phenotype in our high-throughput microscopy pipeline. Shown alongside the gene hits outlined in Fig. 1 are the gene products for each hit. Download TABLE S1, XLSX file, 0.1 MB. Copyright © 2017 French et al. 2017 French et al. This content is distributed under the terms of the Creative Commons Attribution 4.0 International license.

10.1128/mBio.01977-16.2 DATA SET S1  Morphological features collected for each hit gene, which were used in a principal-component analysis, shown in Fig. 1. Download DATA SET S1, XLSX file, 0.5 MB. Copyright © 2017 French et al. 2017 French et al. This content is distributed under the terms of the Creative Commons Attribution 4.0 International license.

**FIG 2:**
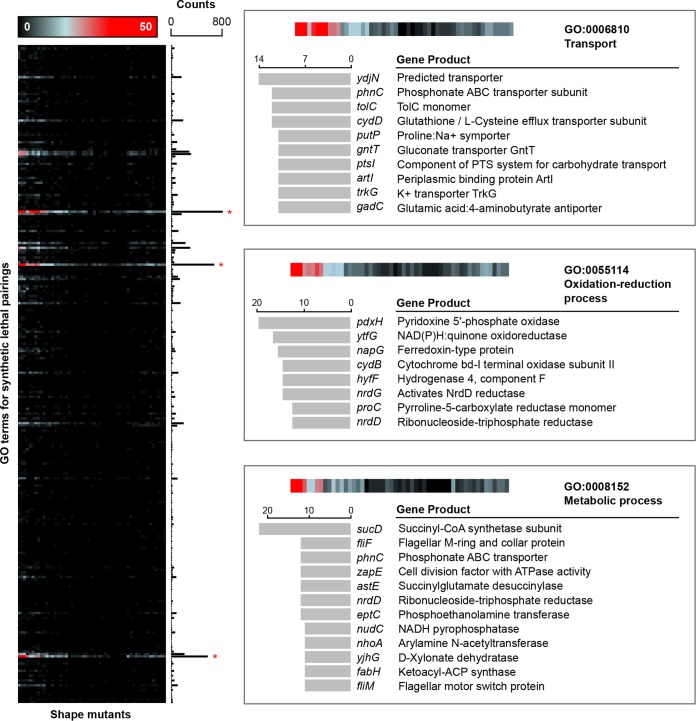
Genome-scale analysis of *E. coli* genes that interact with shape-perturbing genes. The left panel shows a heat map for synthetic lethal interactions and a frequency of occurrence histogram for biological processes, organized by GO terms. Synthetic lethal interactions were identified using the method of Côté et al. (17), with annotations identified using EcoCyc pathway-tools software (19). (GO terms were generated from our 1,373 synthetic lethal genes, with many genes having multiple corresponding biological process terms.) Transport, redox, and metabolic processes displayed strong enrichment and are highlighted with red stars. Each of these processes is further elaborated in the panels to the right, highlighting the most frequently occurring synthetic lethal interactions and their corresponding gene products. Interestingly, many transport genes are closely linked with respiration and proton symporting, such as *cydD*, *putP*, and *trkG*. Overall, this demonstrates an intricate link between cell shape, transport, and oxidation-reduction processes in *E. coli*.

In all, 60 deletions corresponding to the strongest shape defects were chosen as query genes in a synthetic genetic array, 7 of which did not conjugate well using the methodology of Côté et al. (17). This resulted in 53 shape genes of interest from Table S1 being systematically crossed (5, 6) with the Keio collection, generating 1.7 × 10^7^ double deletion mutants (see Data Set S2 in the supplemental material) and a synthetic lethal interaction network (see Fig. S3 in the supplemental material). We noticed that mutants with more extreme shape variations (for example *pdxH*, *rodZ*, *envC*, and *gpmM* mutants) did not mate correctly under conventional conditions and required mating at room temperature for an extended period of time. When the genes underlying synthetic lethal interactions were enriched using Gene Ontology (GO) terms for biological processes, transport and oxidation-reduction processes were overrepresented (Fig. 2; Fig. S3). This was based on a frequency of occurrence histogram for each GO term, with ontologies accessed using EcoCyc (18) pathway-tools software (19). Interestingly, the TolC outer membrane channel is present with high frequency, implicating a relationship between drug efflux system effectiveness (Acr, Mdt, Ent, Emr, and Mac) and cell shape. The predicted symporter YdjN, which occurs most frequently, is further implicated in both l-cysteine transport and oxidative stress response (20), biological processes coincidentally enriched in Fig. 2. Indeed, genes involved in redox processes were often respiration linked, such as in the cases of *ytfG*, *napG*, *cydB*, *nrdD*, and *nrdG*. This is particularly curious given that membrane shape/curvature plays a role in membrane-associated protein function (21, 22), especially given the potential relationships between drug efficacy and cellular respiration (23, 24). There is, however, a limited understanding of the link between cell shape (or surface area) and respiration. We witnessed a remarkable preponderance of interactions of shape genes with those involved in respiratory functions. For instance, in the work by Côté et al. (17), 82 nutrient stress genes were crossed with the Keio collection and resulted in fewer than 200 synthetic lethal interactions with genes involved in redox processes, while in contrast, about 800 such interactions were observed for 53 shape-perturbing genes (Fig. 2).

10.1128/mBio.01977-16.3 DATA SET S2  Nodes and edges used to generate the three-dimensional (3D) network map shown in Fig. S3. Shown alongside the nodes are the normalized colony sizes of the double deletion pairing, labeled here as “Distance.” Download DATA SET S2, XLSX file, 0.1 MB. Copyright © 2017 French et al. 2017 French et al. This content is distributed under the terms of the Creative Commons Attribution 4.0 International license.

10.1128/mBio.01977-16.5 FIG S2  Principal-component analysis (PCA) loading biplot. This accompanies the PCA plot in Fig. 1 and shows the first three principal-component scores on the major axes, along with morphological descriptor eigenvectors. This was generated using the *pca3d* package in the R statistical programing language (R. Ihaka and R. Gentleman, J Comput Graph Stat 5:299–314, 1996). Download FIG S2, PDF file, 0.3 MB. Copyright © 2017 French et al. 2017 French et al. This content is distributed under the terms of the Creative Commons Attribution 4.0 International license.

10.1128/mBio.01977-16.6 FIG S3  Network interaction map for 1,373 synthetic lethal pairings. Lethal interactions were determined by the method of Côté et al. (mBio 7:e01714-16, 2016, https://doi.org/10.1128/mBio.01714-16), with a 2.5-σ cutoff, and shown in the manner of French et al. (Mol Biol Cell 27:1015–1025, 2016, https://doi.org/10.1091/mbc.E15-08-0573) using BioLayout Express 3D (A. Theocharidis, S. van Dongen, A. J. Enright, and T. C. Freeman, Nat Protoc 4:1535–1550, 2009, https://doi.org/10.1038/nprot.2009.177). A Markov clustering method was used to identify clusters of genes within the map, with six distinct clusters dominating. Nodes were sized based on the number of adjacent edges. Each of the interactions in these clusters was assessed for the prevalent GO terms, which are shown alongside the map. Interestingly, there is high connectivity within the network, mainly with respect to transport and redox processes. Download FIG S3, TIF file, 1.9 MB. Copyright © 2017 French et al. 2017 French et al. This content is distributed under the terms of the Creative Commons Attribution 4.0 International license.

Probing the Keio collection for gene deletions resulting in shape defects unexpectedly yielded a wealth of uncharacterized genes. Connecting these genes with roles in cell shape may aid in downstream functional classification, particularly when simultaneously profiling their genetic interactions. Overall, genes involved in shape maintenance seem to be inherently linked to biological processes such as transport and oxidation and reduction, but further work is required to clarify the nature of the interactions observed. Of particular interest is whether physical perturbations such as packing geometry or curvature variations (21) accompany shape defects. Nonetheless, the research presented here expands our understanding of the complicated biology that defines bacterial shape. Furthermore, the high-content microscopy platform described herein is highly amendable to any bacterial genomic or chemical library.
