# Supplementary material for: Bacteria Getting into Shape: Genetic Determinants of E. coli Morphology
Source: mBio. 2017 Mar 7;8(2):e01977-16. doi: 10.1128/mBio.01977-16 (PMC5340871; doi:10.1128/mBio.01977-16)
Supplement: TEXT S1 [file mbo001173215s1.docx]

**Culture conditions and bioactive molecule sources.** *Escherichia coli* K-12 BW25113 and the Keio collection (1) were cultured in Luria-Bertani (LB) broth at 37ºC in standard size 96-well microplates in duplicate. Overnight cultures were subcultured into fresh medium using a RAININ Liquidator 96 system (Mettler-Toledo Inc., Mississauga ON, CAN), and grown to approximate mid-exponential growth phase (about five hours) in preparation for high-throughput microscopy. Depending on available equipment, any liquid handling system can prepare the cultures and microscopy plates outlined in this experiment.

**Microscopy preparations.** To maintain morphological integrity over time, 10 µL of culture was transferred into a 96-well microplate containing 90 µL of 2% glutaraldehyde in 25 mM HEPES (pH 6.8). Plates were then kept at 4ºC for 1-3 hours to limit *de novo* syntheses of cell envelope structures during coating with the glutaraldehyde polymer. Twelve microliters from each plate were then transferred into 384-well microplates with 0.17 µm optically clear film bottoms (Matrical Bioscience, Spokane WA, USA), along with five microliters of 1.5% (w/v) freshly filtered nigrosin as a negative stain. Plates were flushed with N_2_ gas to remove any bubbles, and then liquid in the microplates was gently removed by incubating plates at 50ºC in a humidity-controlled incubator. The cooling and heating steps did not introduce morphological artefacts, based on tests with positive and negative control strains.

**High-throughput microscopy.** Microplates containing fixed cells were painted with a layer of immersion oil across the bottom. Plates were visualized using light microscopy with a Nikon Eclipse TE200 inverted microscope base, which was coupled with a Prior ProScan II motorized stage system and Z focus sleeve. Samples were imaged with a Hamamatsu ORCA Flash 2.8 camera coupled with a 0.6x C-mount adapter, using a 100x Nikon CFI Plan Fluor DLL objective. The stage and focus were driven by a Prior ProScan II controller and interfaced with the open-source microscopy package Micro Manager (2). When autofocusing was used, we implemented an autofocusing system utilizing the Micro Manager JAF(H&P) system. This utilizes Sobel operators to determine image sharpness in a 2-step iterative process of course (rough, long distance) and fine (small distance) focusing. Depending on the microscope used, this will change with the preference of the user, particularly with modern laser-based focusing hardware. Typically, at least 200 cells were counted per well.

**Image analysis and determination of abnormal morphology.** Images were background subtracted with a 50-pixel rolling ball radius, converted to threshold images in batch using ImageJ and the Otsu threshold algorithm, then subjected to a watershed function. Cell features were quantified using particle analysis, quantifying: Cell area, perimeter, major axis and Feret axis, minor axis and minor Feret axis, circularity, aspect ratio and roundness, and solidity. All downstream calculations were done in the R statistical programming language (3). Principal component analysis (PCA) was used as a means of dimensionality reduction for screening purposes. Most variances are explained primarily from the first three principal components (Fig.1), allowing for 3D representations of points in principal component space. A single cutoff value to determine abnormal cell shape is not appropriate here, as the cutoff would vary for each principal component. As such, we use an ellipsoid to account for a three standard deviation cutoff in each of the three dimensions contributing to the sample variance. Points falling outside this ellipsoid are considered to be abnormal for screening purposes.

**Synthetic lethal array and GO term enrichment.** The synthetic lethal array for our query genes was generated according to the method of Côté et al (4). Using this array, gene ontology (GO) terms were obtained for each of the synthetic lethal interactions generated from conjugation with our morphology mutants. This was done in batch using *pathway-tools* in EcoCyc (5, 6), and GO term counts were obtained for each query gene. This allowed us to look for overrepresentation amongst the GO terms tightly linked to abnormal bacterial morphology based on synthetic lethal interactions.

**References**

1. **Baba T**, **Ara T**, **Hasegawa M**, **Takai Y**, **Okumura Y**, **Baba M**, **Datsenko KA**, **Tomita M**, **Wanner BL**, **Mori H**. 2006. Construction of *Escherichia coli* K-12 in-frame, single-gene knockout mutants: the Keio collection. Mol Syst Biol **2**:2006.0008.

2. **Edelstein A**, **Amodaj N**, **Hoover K**, **Vale R**, **Stuurman N**. 2010. Computer control of microscopes using µManager. Curr Protoc Mol Biol **Chapter 14**:Unit14.20.

3. **Ihaka R**, **Gentleman R**. 1996. R: A Language for Data Analysis and Graphics. J Comput Graph Stat **5**:299–314.

4. **Côté J-P**, **French S**, **Gehrke SS**, **MacNair CR**, **Mangat CS**, **Bharat A**, **Brown ED**. The genome-wide interaction network of nutrient stress genes in *Escherichia coli*. MBio Accepted. mBio01714–16.

5. **Karp PD**, **Latendresse M**, **Paley SM**, **Krummenacker M**, **Ong QD**, **Billington R**, **Kothari A**, **Weaver D**, **Lee T**, **Subhraveti P**, **Spaulding A**, **Fulcher C**, **Keseler IM**, **Caspi R**. 2015. Pathway Tools version 19.0 update: software for pathway/genome informatics and systems biology. Brief Bioinformatics bbv079.

6. **Keseler IM**, **Mackie A**, **Peralta-Gil M**, **Santos-Zavaleta A**, **Gama-Castro S**, **Bonavides-Martínez C**, **Fulcher C**, **Huerta AM**, **Kothari A**, **Krummenacker M**, **Latendresse M**, **Muñiz-Rascado L**, **Ong Q**, **Paley S**, **Schröder I**, **Shearer AG**, **Subhraveti P**, **Travers M**, **Weerasinghe D**, **Weiss V**, **Collado-Vides J**, **Gunsalus RP**, **Paulsen I**, **Karp PD**. 2013. EcoCyc: Fusing model organism databases with systems biology. Nucleic Acids Res **41**:D605–12.
