# Supplementary figures and images for: Bacteria Getting into Shape: Genetic Determinants of E. coli Morphology
Source: mBio. 2017 Mar 7;8(2):e01977-16. doi: 10.1128/mBio.01977-16 (PMC5340871; doi:10.1128/mBio.01977-16)

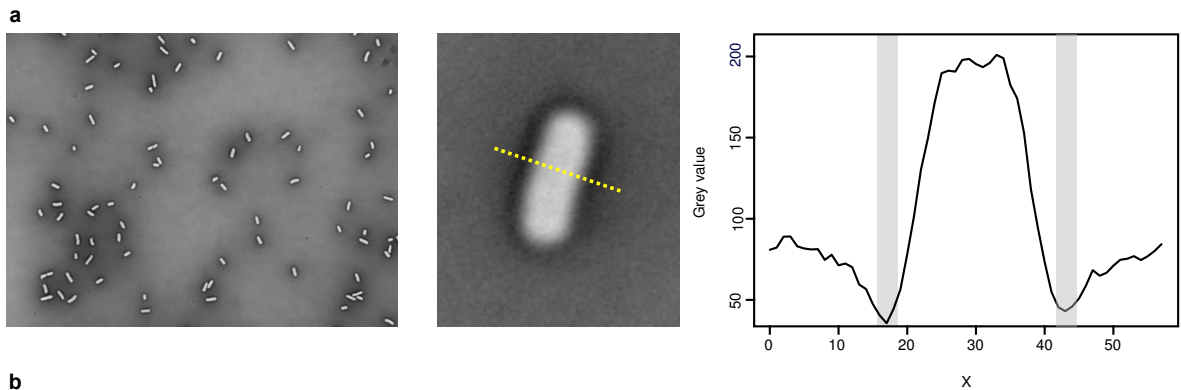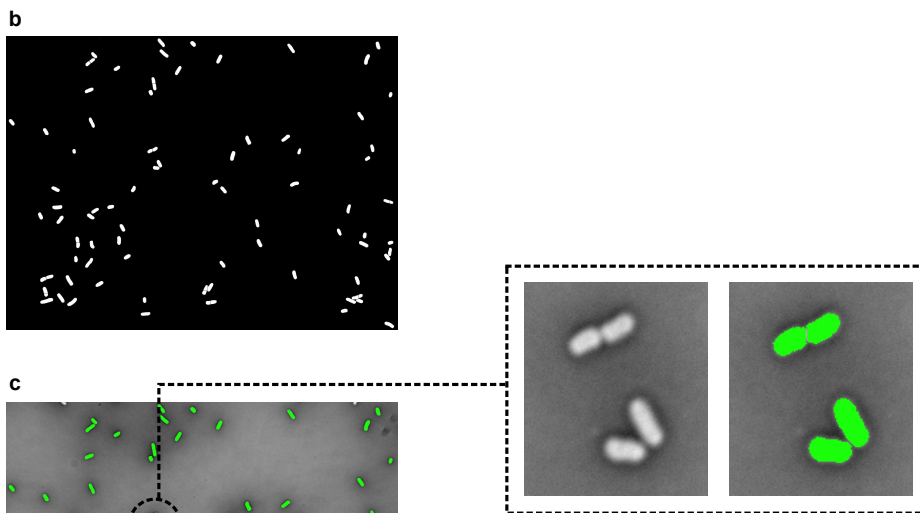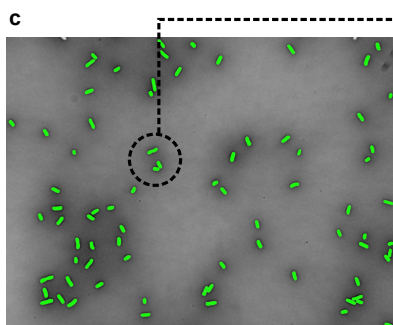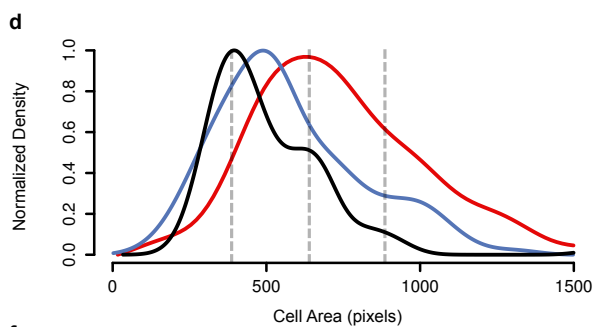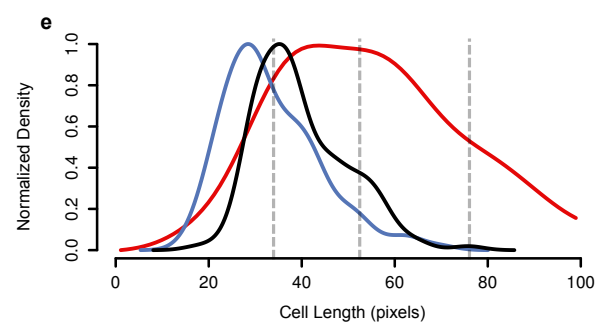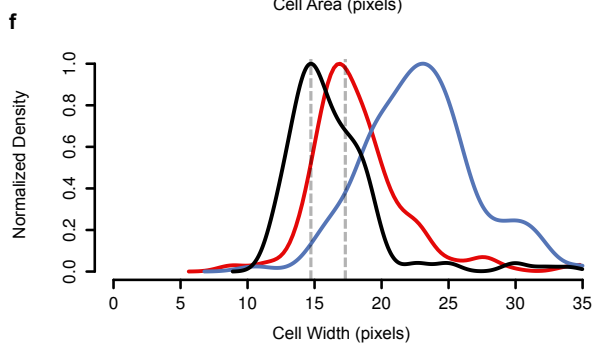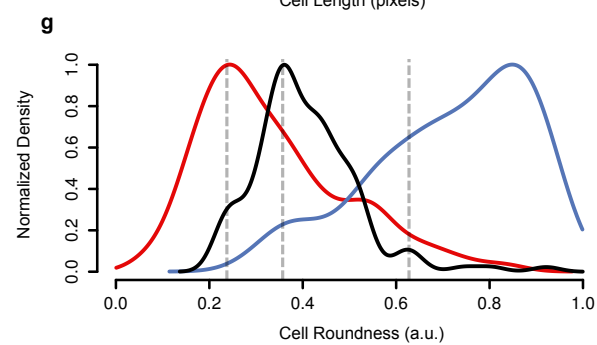

Supplement: FIG S1 [file mbo001173215sf1.pdf]

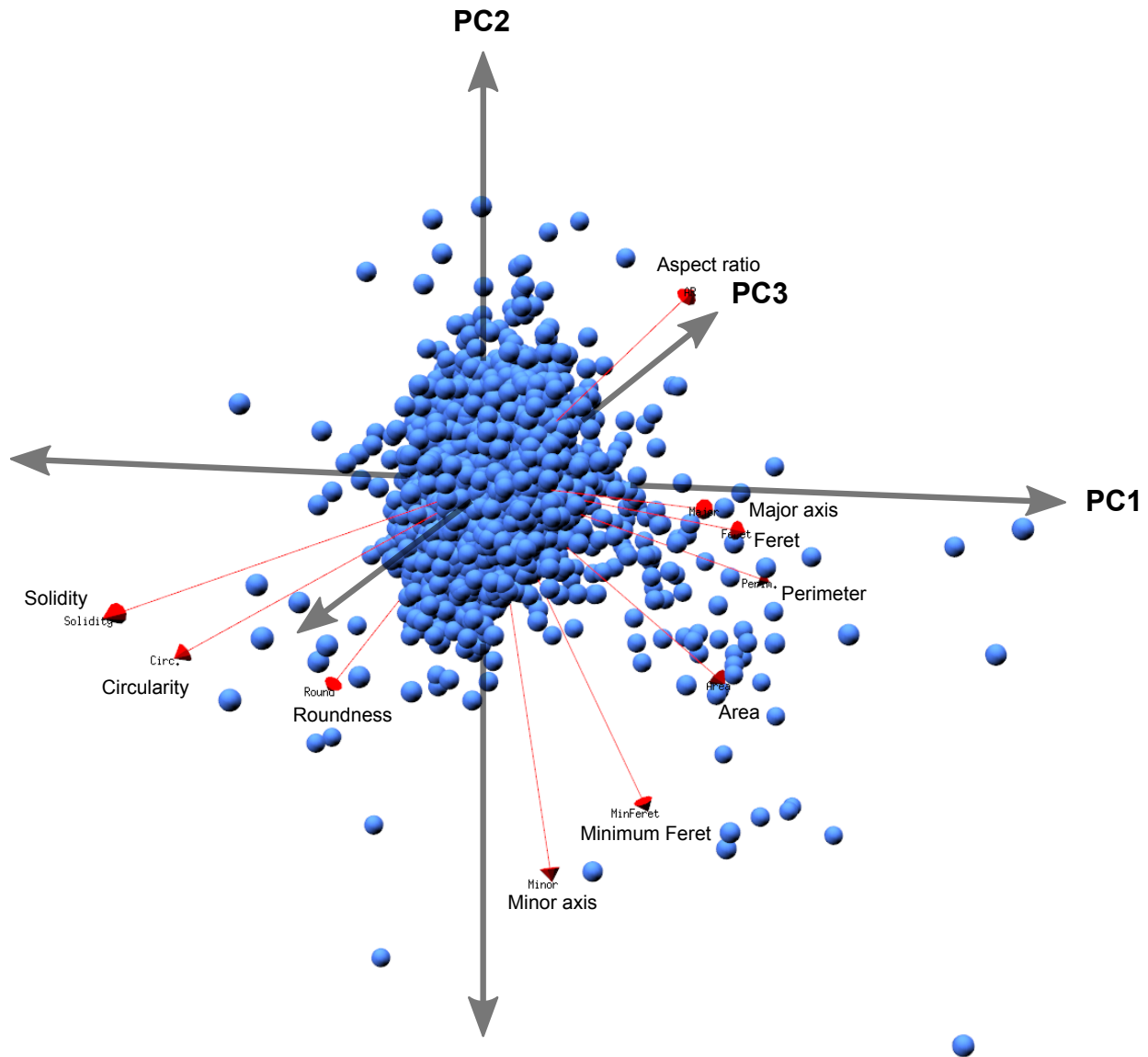

Supplement: FIG S2 [file mbo001173215sf2.pdf]

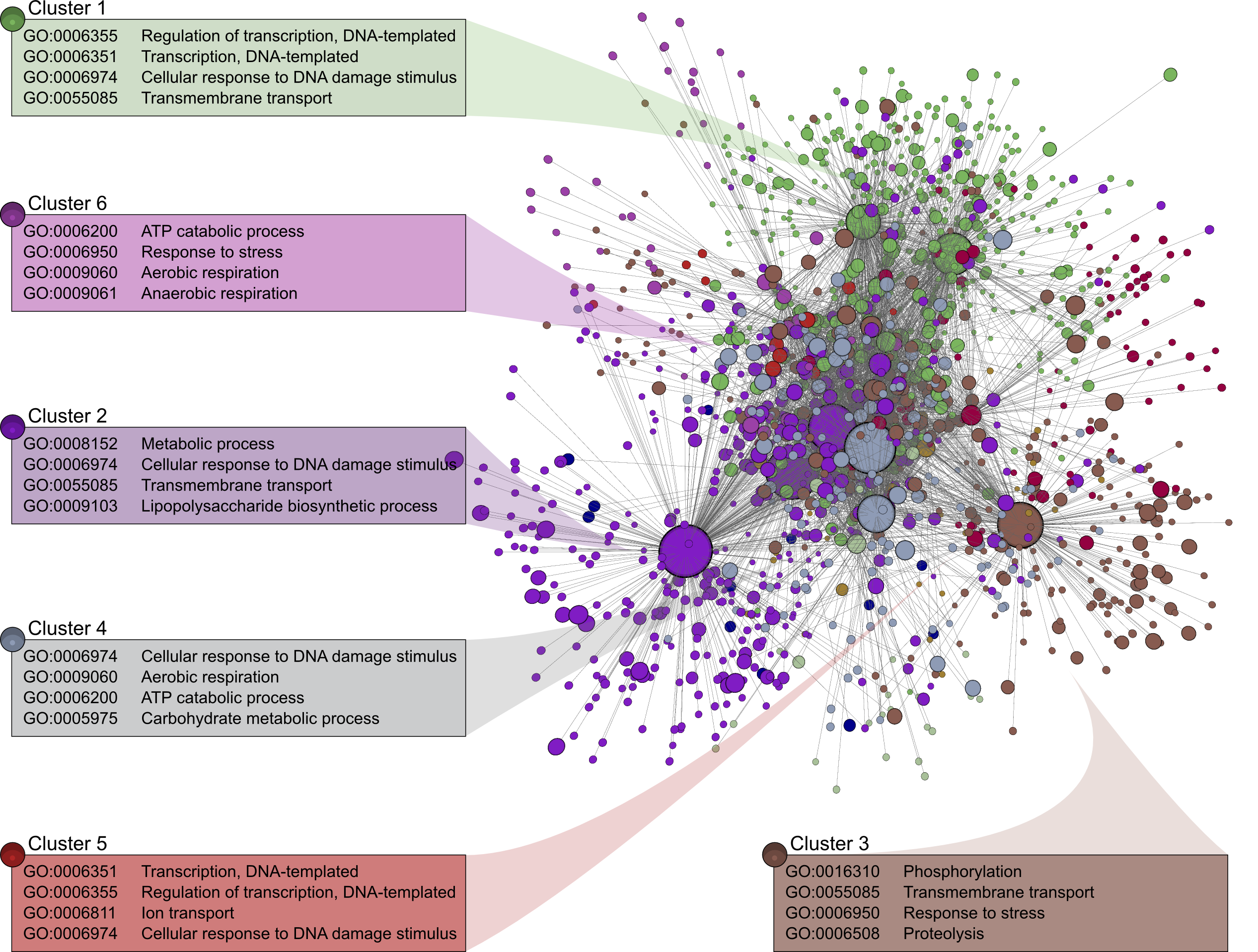

Supplement: FIG S3 [file mbo001173215sf3.tif]
